# Feasibility of Location-Aware Handover for Autonomous Vehicles in Industrial Multi-Radio Environments

**DOI:** 10.3390/s20216290

**Published:** 2020-11-05

**Authors:** Yi Lu, Mikhail Gerasimenko, Roman Kovalchukov, Martin Stusek, Jani Urama, Jiri Hosek, Mikko Valkama, Elena Simona Lohan

**Affiliations:** 1 Department of Electrial Engineering, Tampere University, 33720 Tampere, Finland; gerasimenkoma89@gmail.com (M.G.); roman.kovalchukov@tuni.fi (R.K.); xstuse01@vutbr.cz (M.S.); jani.urama@tuni.fi (J.U.); mikko.valkama@tuni.fi (M.V.); elena-simona.lohan@tuni.fi (E.S.L.); 2 Department of Telecommunications, Brno University of Technology, 616 00 Brno, Czech Republic; hosek@feec.vutbr.cz

**Keywords:** dead reckoning, geometry-based positioning, indoor industrial environments, location-aware handover, mmWave communications, multi-radio access, radio positioning

## Abstract

The integration of millimeter wave (mmWave) and low frequency interfaces brings an unique opportunity to unify the communications and positioning technologies in the future wireless heterogeneous networks (HetNets), which offer great potential for efficient handover using location awareness, hence a location-aware handover (LHO). Targeting a self-organized communication system with autonomous vehicles, we conduct and describe an experimental and analytical study on the LHO using a mmWave-enabled robotic platform in a multi-radio environment. Compared to the conventional received signal strength indicator (RSSI)-based handover, the studied LHO not only improves the achievable throughput, but also enhances the wireless link robustness for the industrial Internet-of-things (IIoT)-oriented applications. In terms of acquiring location awareness, a geometry-based positioning (GBP) algorithm is proposed and implemented in both simulation and experiments, where its achievable accuracy is assessed and tested. Based on the performed experiments, the location-related measurements acquired by the robot are not accurate enough for the standalone-GBP algorithm to provide an accurate location awareness to perform a reliable handover. Nevertheless, we demonstrate that by combining the GBP with the dead reckoning, more accurate location awareness becomes achievable, the LHO can therefore be performed in a more optimized manner compared to the conventional RSSI-based handover scheme, and is therefore able to achieve approximately twice as high average throughput in certain scenarios. Our study confirms that the achieved location awareness, if accurate enough, could enable an efficient handover scheme, further enhancing the autonomous features in the HetNets.

## 1. Introduction

Wireless communications and mobile networks are inherently integrated into the daily activities of every business sector, ranging from academic events to industrial operations. With the paradigm introduced by ITU, known as “Always Best Connected” (ABC) [1], the 3rd generation partnership project (3GPP) has proposed the coordinated multi-point (CoMP) operation in [2], where coordinated transmission and reception applies between macro cell(s) and micro cell(s) of heterogeneous networks (HetNets) to provide seamless, quality of service (QoS)-aware media services to the active user equipment (UE), including Internet of Things (IoT) devices and/or mobile vehicles therein. Furthermore, dual connectivity (DC) has been proposed in [3], where any arbitrary UE enjoys radio resources provided by access point (AP) with the same/different radio access technology (RAT) under non-ideal backhaul. To further exploit the benefits of DC from the perspectives of user throughput and mobility enhancement within small cell networks (SCNs), multi-radio dual connectivity (MR-DC) [4] grows to become a feasible solution for the cooperation/co-existence of 4G long term evolution (LTE) and 5G new radio (NR), facilitating the convergence of communications and positioning solutions as well as enabling the autonomous function for the connected vehicles. As an extension of DC, MR-DC suggests that the UE can be configured to maintain multiple connections [5] and utilize resources of multi-RAT enabled networks, i.e., the HetNets.

In the context of multi-RATs and SCNs, seamlessly handing over to or selecting a network/RAT, which provides the best possible QoS to the UE, remains a crucial enabler of a self-organized communication system, in which the connected vehicles/robots are capable of performing efficient handover in a self-organized manner via the obtained location awareness, such that the throughput as well as the link quality are jointly optimized. Apart from the IEEE 802.21 multimedia-independent handover (MIH) standard that supports a QoS-based handover within the HetNets, several works [6,7,8,9,10,11,12,13,14,15,16,17,18] investigated the location-aware handover (LHO) and/or location-aware network selection (LNS) strategy ensuring that the mobile users have access to the required media service via the best available network. The objective of our work is therefore to investigate the feasibility and benefits of such efficient handover decision making schemes (i.e., the LHO) when integrating the positioning solutions. Furthermore, as a result of the proliferation of industrial vehicles [19], we believe that the LHO algorithm will not only be beneficial to the conventional mobile users, but also it will be desirable for vehicle/robot-based industrial Internet of things (IIoT) applications where multi-Gbps throughput is required for data offloading/exchange (e.g., virtual reality [20]) with one or several millimeter wave (mmWave) APs.

In this paper, we describe an efficient LHO scheme for a multi-RAT robotic platform in an indoor multi-radio environment. Thereafter, an experimental study is performed to evaluate the concept feasibility and demonstrate the achievable performance. In our measurement setup, one mmWave AP and one centimeter wave (cmWave) AP (WiFi at 2.5 GHz) are deployed and utilized. Furthermore, we construct a 3D line-of-sight (LoS) map for the area of interest where the LHO algorithm is tested. This map indicates the area where the robot has the LoS communications with the mmWave AP. Then, by considering the available location-related measurements (LRMs) (to be discussed in Section 2.2) at the robot side, we formulate a geometry-based positioning (GBP) algorithm and evaluate the corresponding achievable positioning accuracy in theory.

Although the theoretical positioning accuracy is shown to be promising, the experimental outcomes with a standalone GBP are hardly satisfactory due to the inaccuracy and instability of LRMs, as we discuss in Section 5.2. However, by combining the GBP and the dead reckoning (DR) methods, the experimental results corroborate that with the LHO scheme a handover can be performed right before the robot loses its LoS connection with respect to (w.r.t.) the mmWave AP. Hence, the LHO scheme guarantees higher throughput for the robot than the conventional received signal strength indicator (RSSI)-based handover approach. The key contributions of our paper are summarized as follows:

- we formulate and analyze a positioning algorithm, GBP, based on the angle measurements from one mmWave AP enabling 2D positioning in the 3D environment;
- we propose an efficient LHO handover scheme that utilizes both the GBP positioning algorithm as well as the DR method;
- we study and test the proposed handover scheme using a multi-RAT prototype robot developed by our group, and we assess the statistics of RSSI, latency, and estimated robot locations.

The rest of the paper is organized as follows. In Section 2, we systematically review the state-of-the-art in LHO/LNS, which is followed by the introduction of the problem formulation. Section 3 outlines the utilized multi-RAT robotic platform as well as the scenario of interest for conducting the LHO. In Section 4, we describe the constructed 3D map for our simulation campaign together with the proposed positioning algorithm and handover scheme. Section 5 provides a discussion on the simulation results of the theoretical positioning accuracy and the experimental results related to the handover schemes. Finally, Section 6 wraps up this paper with a conclusion.

## 2. State-of-the-Art Overview and Problem Formulation

Here, an overview of both the handover schemes and the positioning techniques is given followed by the state-of-the-art and the problem formulation.

### 2.1. Overview of Handover Schemes

The implementation of a handover procedure is necessary in any wireless network because of the mobility of the users within. However, such implementation depends on various factors including the average coverage of the serving entities e.g., AP or base station (BS), the trajectory and the velocity of the users, the multiple access scheme (orthogonal or non-orthogonal multiple access), carrier frequency and the type of the network (mmWave based or cmWave based, Homogeneous Networks or HetNets, etc.). Handovers are also classified based on the decision entity, decision criteria, and performance metrics. A comprehensive overview of handover schemes can be found in [21,22], where the authors paid particular attention to the so-called vertical handover. The latter is a switch between different RATs or standards, which is a key mechanism to enable the HetNets. In cellular networks, handover decisions are usually made in a centralized manner. In other words, the handover is in general carried out based on the downlink RSSI measurements together with the corresponding hysteresis and timer, during which the RSSI of the target cell should be higher than the RSSI of the original cell. However, in vertical handover it is not always efficient to use traditional metrics [23].

In addition, handovers can be classified based on the target performance. For example, a handover scheme allowing to switch the cell without a service interruption (minimized packet loss) is called “seamless” handover, while in “fast” handover the latency of packets is minimized. In the case of mmWave access, additional issues arise: due to high channel penetration loss, the zone where a handover may be performed without session interruption is usually very small [24]. This problem also appears in the case of LoS–non line-of-sight (NLoS) boundary crossing, where mmWave signal level drops significantly and rapidly, and the traditional RSSI-based handover schemes cannot react fast enough.

### 2.2. Overview of Positioning Techniques

In general, positioning techniques are categorized in several different ways [25,26], wherein each category may differ in terms of various physical media signal used (e.g., sound, light, or radio signal), or different principles of obtaining the location of the target of interest (e.g., proximity, fingerprinting, or multilateration). Alternatively, the positioning techniques stem from a specific positioning system, which can be classified into two groups: self-positioning systems and remote-positioning systems ([27] Ch. 1). While the former is essentially a DR system [28], as it computes the current location based on the previous location by fusing the heading and the distance measurements obtained from the inertial measurement unit (IMU) sensors, the remote-positioning system is referred to as a radio-positioning system, as it relies on the LRMs wherein the relative location information is embedded. Typical LRMs consists of RSSI, time of arrival (ToA), and/or angle of arrival (AoA).

For a radio-positioning system, the acquisition of the LRMs relies on different equipment and/or methods. Owing to the simplicity of acquisition and low-complexity in terms of the needed hardware, RSSI-based positioning remains an attractive approach especially for indoor scenarios ([27] Ch. 11). However, several error sources, such as multi-path and NLoS, impair the stability and prediction quality of the RSSI measurements and therefore may keep the positioning accuracy away from being acceptable by the requirements specified for certain use cases [29]. In addition, the ToA measurements can also be employed for positioning by using a multilateration principle ([27] Ch. 6), such as the global navigation satellite system (GNSS)-based positioning. Conditional on a perfect synchronized clock among the UE and AP, the ToA-based positioning is capable of achieving much higher accuracy than the RSSI-based positioning especially at higher carrier frequencies and over wider bandwidths [30]. However, due to inherent clock offset among transmitters and receivers, certain approaches are required to compensate for the synchronization errors [31,32]. Last but not least, there is also the AoA-based positioning enabled by antenna arrays or directional antennas ([27] Ch. 9), such that the direction from which the signal arrives at the receiver (i.e., the AP in a network-centric positioning system) is known. Hence, a location estimation is obtained by applying the multi-angulation principle ([33] Ch. 2). Under both LoS and NLoS propagation, the antenna orientation at the receiver side must be known or estimated to make the AoA measurements reasonable for positioning.

Besides the positioning principles for different LRMs, various algorithms can be applied to learn the location estimates based on one or multiple LRMs ([27] Ch. 2, [34,35]). In this work, the robot location is acquired by combining the radio-positioning techniques (for the estimation of initial location of the robot) and the self-positioning (for subsequent positioning) by utilizing the outputs of the on-board sensors to predict the moving distance and the direction of the robot at each time instant.

### 2.3. State-of-the-Art in the Location-Aware Communications and Handover Schemes

A location-aware adaptive communication system has been investigated in ([33] Ch. 9), where adaptive modulation and coding (AMC) together with location information was cooperatively combined in order to achieve macro diversity. It was suggested in [33] that wireless communications can benefit from a proactively updated location information of the UEs. This is because location-aware communication systems are capable of predicting more precisely the channel state information (CSI) at the transmitter for adaptive beamforming as compared to location-unaware communication systems. With a perfect knowledge of location and a fingerprint database, a location-aware system showed decent improvements in terms of the mean capacity over pure CSI-based systems especially for the applications with long feedback delays and large channel variations. However, the advantage of a location-aware system may not hold when positioning error increases above a certain threshold. In practical situations, it is likely that positioning errors may be excessive for the UE to benefit from a location-aware communication mechanism.

As an indispensable component of the handover process, network selection algorithms have been studied with location awareness, i.e., LNS. In [6], a TCP/IP based LNS architecture was proposed on top of IEEE 802.21 MIH standard. Specifically, location awareness of mobile users as well as the available network information were monitored periodically by a location & network monitor (LNM). The former was then exploited to predict the distances to the currently available networks, user trajectory, as well as the mobility patterns, for designing the LNS algorithms. Despite better handover performance than without location awareness, the impact of location errors of the mobile users towards the LNS was not considered therein. Additionally, cloud-based network selection for vehicular networks was proposed in [7] for leveraging the rich computing power and data storage of a cloud computing server. The efficiency of the aforementioned network selection algorithms stemmed from offloading complex computations to a geographically distributed cloud server, which in turn provided better decision-making in the network selection, based on a broader network information. Moreover, a fast convergence algorithm for solving a coalition formation game was proposed in [7] to enable optimization over larger scale networks and practical implementation.

Works on LHO under different scenarios can be found in [8,9,10] and the references therein. Taking into account the information of both user location and network load, the authors in [8] studied the LHO scheme in multi-cell networks. Two metrics have been utilized to optimize the handover process, namely, the angle of handover and the load-balancing index. The former suggests that the moving direction of the UEs can be computed based on the previous location estimates by the GNSS, which is then exploited to predict the most likely target cell that the UE will connect to. Further, the consideration of the load balancing index is to ensure that the QoS of the UEs does not drop if the target cell is already heavily loaded. The simulation results in [8] showed that the proposed LHO algorithm outperforms the conventional one, especially for UEs with high-but-constant velocity and UEs that make turns less frequently.

Similarly, relying on the location estimation provided by GNSS, authors of [9] proposed an efficient network selection scheme based on not only the estimated location of the users (via GNSS technique) but also maps reflecting the channel quality information (i.e., the average of signal strength) as well as the traffic load. Due to the rapid changes of traffic load, frequent updates of such information are therefore necessary which poses a stringent requirement in terms of the latency and throughput of back-haul link of the HetNets. However, the proposed schemes may suffer severe degradation when GNSS-based positioning is disabled or provides low-accuracy location estimates, which can be the practical cases especially in the urban macro scenario.

With the proxy mobile IPv6 (PMIPv6) in mind, the authors of [10] investigated several handover schemes, such as fast handover for PMIPv6 (FPMIPv6), smart buffering, and low-latency handover, and compared the corresponding handover performance with the proposed location-aware fast PMIPv6 (LA-FPMIPv6). In particular, the FPMIPv6 aims at reducing the packet loss by scanning and detecting the network status of all APs around the source AP. The handover is thus prepared once the RSSI of the source AP falls below a certain threshold. Similarly, the smart-buffering scheme, which also predicts the handover based on the RSSI, was proposed in [10] to reduce the packet loss. The main difference between smart-buffering scheme and FPMIPv6 lies in the fact that in the FPMIPv6 the UE scans the surrounding APs once handover decision is made, whereas for the smart-buffering scheme, the target AP, with which the UE is connected, searches for the source AP to retrieve the buffered packets. The resulting handover latency is the same as with the PMIPv6, but smart buffering enjoys a lower packet loss. Furthermore, by omitting the UE’s authentication procedure, low-latency handover was also proposed in [10] to reduce the packet loss. However, due to the fact that only RSSI has been considered as the parameter, the handover cannot be predicted precisely whenever the measured RSSI is considerably different from the actual values. Hence, the LA-FPMIPv6 was proposed to perform a handover based on both the location information of the UE as well as the RSSI. Hence, both the handover timings and the next AP to which the UE will connect can be predicted more precisely than in the aforementioned algorithms, and therefore it achieves enhanced performance in terms of the reduced signaling cost, buffering cost, and handover latency.

As key components in reducing the network latency and increasing the link robustness, control/user plane separation (CUPS) [36] together with MR-DC have been employed to improve the handover performance in mmWave HetNets. The authors of [11] proposed a prevenient handover scheme with radio resource control signalling duplication and master-secondary switch in order to reduce the handover failure rate and service interruption time. Similarly, for a CUPS network, the authors of [12] presented a seamless handover scheme by introducing a handover-assisted micro evolved NodeB (HO A-eNB) in the overlapping area. Such HO A-eNB maintains a continuous connectivity with the UE throughout the handover process, resulting in improved success probability of handover. However, deploying such HO A-eNBs in all the possible overlapping areas may incur prohibitive costs as well as posing a stringent requirement on the backhaul link capacity of the network.

By integrating the mmWave networks with the sub-6GHz networks for better link robustness and higher throughput, the benefit of performing handover with location awareness of the UE has been investigated in [13,14,15]. In [13], the location and velocity information (mobility information) of the UE was exploited to optimize the overall network performance, such as reducing the number of handovers as well as enhancing the user QoS. However, similar to the aforementioned works, the way of acquiring a perfect knowledge of the mobility information of the UE was not clearly specified. Moreover, the importance of location information has been investigated in [14,15]. By exploiting the available CSI at sub-6 GHz bands, the authors in [14] proposed to apply a machine learning method to predict the location information of the UE, which is then utilized to achieve faster handover. The results showed that with location awareness, the UEs enjoy higher spectral efficiency than with the conventional handover scheme. Furthermore, with the objective of allowing the mmWave networks to operate at scale, the authors in [15] shared their thoughts on the scalability challenges in mmWave networks and also presented positioning algorithms (network-based and device-based) for achieving high accuracy of location awareness. Such positioning algorithms can be not only employed to enable location-based services, but also help reduce the handover costs and make optimized handover decisions.

In addition to the handover schemes based on location- and mobility-awareness, other contextual information, such as quality of experience (QoE) and radio environment map (REM), has also been employed and exploited to improve the handover performance. Targeting at improved UE’s QoE, the authors in [16] have presented a Q-learning based algorithm for vertical handoff in the HetNets. Via continuous interaction with the environment, an optimized handoff strategy is achieved, i.e., the QoE is maximized based on created mappings between QoS metrics and achieved subjective experience on the side of UEs). Meanwhile, in [17], the REM as well as the UE trajectories are considered to predict the quality of network connectivity that is then exploited for handover process. It is seen that the handover performance improves when the time-to-trigger, the UE velocity, and the location error are low. However, the addressed carrier lies in sub-6 GHz band, and REM of mmWave APs was not considered.

Further work using REM for handover can be found in [18], where positioning and radio maps have been combined together for intra-frequency handover. Applying two location prediction methods, the handover decision was made by determining the BS that may offer the highest reference signal received power (RSRP) based on the radio map. However, unconditionally switching to the BS with the highest RSRP might result in frequent handovers and a high ping-pong rate [11]. Contrary to the handover that chooses the BS with the highest RSRP, the authors of [37] proposed to perform a handover only if the signal-to-interference-plus-noise ratio (SINR) at the UE side is lower than a certain threshold over the entire time-to-trigger period. Accordingly, a handover is not triggered even if the RSSI from the serving BS is not the highest, thus, reducing the unnecessary handovers. However, the impact produced by the prediction error of the RSSI was not considered therein, which is the case in practical HetNets.

In addition to service-based architecture (SBA) and CUPS, both the core network and the radio access network (RAN) of 5G NR support network slicing (NS) [38] as another enhancement compared to evolved packet core (EPC) ([39] Ch. 6). Essentially, NS serves as the key enabler for the deployment of multiple virtual networks operating on a shared physical network/infrastructure, and each virtual network can therefore be configured to support different specific network functions indicated by the SBA. Consequently, a RAN slicing-based handover scheme was proposed to provide better UE QoS during a handover [40]. Based on the envisioned hierarchical control model, a handover was triggered based on the current link quality as well as the network condition. More importantly, the handover decision not only indicated the target BS, but also suggested a target RAN slice that better satisfied the UE QoS. Given a highly virtualized network, such a handover scheme is capable of more flexible resource utilization and allocation. However, the performance of such scheme is limited by the available bandwidth/resources of the corresponding RAN as well as the respective propagation condition. In the case of realistic situation, such as, blockage/NLoS, a vertical handover between cmWave and mmWave RATs remains crucial to ensure the reception/decoding of signals from both control and user planes, thus, is more efficient than NS-based handover.

More related works using similar RATs and addressing industrial use cases can be found in [41,42,43]. Specifically, the authors of [41] presented a proactive handover method based on an assessment of the RSSI, the procedure of which remains rather similar to one of the handover methods considered in our work. However, the handover was considered between APs of the same RAT instead of a multi-RAT situation. In the conclusion, the authors also mention that such RSSI-based handover can be improved by considering the user mobility, which is another measure of the location awareness being considered in our work as well. Additionally, the authors of [42] have presented a WiFi/WiGig handover based on the RSSI, no other handover schemes were studied nor investigated therein. Furthermore, the authors of [43] proposed handoff schemes for applications involving the mobility of robots and devices in a real industrial environment. By considering multiple metrics, such as the mobility awareness, RSSI and packet delivery condition, the study showed that the handoff can be triggered with high accuracy and reduced ping-pong effect. Admittedly, handover considering one metric may lead to inaccurate and frequent triggering, therefore, integrating various metrics results in accurate decision making for the handover. In our work, we demonstrate that when location awareness is accurate and reliable enough, the trigger timing for handover can be computed with high accuracy. It is noteworthy that integrating several metrics undoubtedly increases the algorithm complexity and latency in the overall handover process.

Compared with the aforementioned works, our work differs in two major aspects, (i) instead of assuming perfect knowledge of the UE locations/trajectories, we develop a dedicated positioning algorithm (i.e., GBP) to achieve location awareness. In addition, the positioning results and their impact on the handover performance are analyzed and assessed by our simulations as well as practical experiments; (ii) unlike the works supported only by numerical simulation, a multi-RAT robotic platform is employed in our experiments to justify and assess the real-world feasibility of the LHO algorithm.

### 2.4. Problem Formulation

Based on the HetNets structure that is featured with the MR-DC (multi-connectivity [5]), the main objectives of our work are to experimentally assess the feasibility of performing an efficient handover scheme, i.e., the LHO in a multi-radio environment, to evaluate the benefits of such handover over the conventional RSSI-based approaches, and to understand the key factors of maintaining such improvements. Essentially, the LHO benefits from the advantages of both cmWave and mmWave APs within the HetNets that we summarize in Table 1. In particular, the throughput of the robot is augmented by connecting it with the mmWave AP as long as the robot is in the LoS state w.r.t. the mmWave AP. Meanwhile, more robust connectivity can be ensured by handing over to the cmWave AP before the robot loses the LoS connection w.r.t. the mmWave AP. Therefore, the key performance indicators of the LHO are the reliability of the achieved location awareness and the corresponding positioning accuracy.

**Figure 1:**
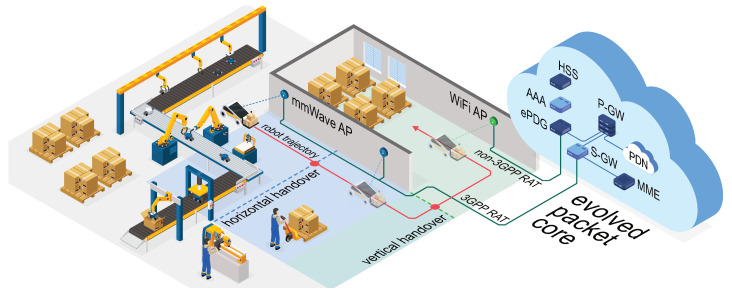
A conceptual figure of the principle of the location-aware handover (LHO) scheme in an industrial multi-radio environment.

To illustrate our technical context, a conceptual figure of the principle pf the proposed handover scheme is developed in Figure 1, where a robot (i.e., a mobile vehicle) is moving within an industrial multi-radio environment while performing certain tasks, such as cargo transportation or video surveillance. To ensure the service quality of the tasks that require wireless connectivity, our objective is to enhance the link robustness while augmenting the throughput throughout the whole robot trajectory. As illustrated by Figure 1, the robot is communicating with the mmWave AP, wherever there is LoS radio connection between them (with a potential horizontal handover, i.e., a switch between the networks of the same RAT in the light blue region), and switches to WiFi (i.e., a vertical handover) before entering the NLoS region of the mmWave AP (see the light green region) based on the available location awareness of the robot, i.e., LHO.

Even though a related topic was investigated in several past works as discussed in Section 2, most of the existing papers report enhanced communication performance by assuming ideal location information for all the UEs/vehicles within the network. In our work, the location information is not assumed to be known perfectly, but rather is estimated via the positioning solutions. Exploiting a laboratory setup, we implement and evaluate the positioning algorithms as well as the LHO scheme utilizing a multi-RAT robotic platform. From the network-level perspective, all data are transferred by the evolved packet core (EPC) with 3GPP RAT (mmWave) stream routed via the serving gateway (S-GW) and packet data network gateway (PGW) to the Internet, represented by the packet data network (PDN). The non-3GPP data is served in the same manner utilizing the evolved packet data gateway (ePDG). At the same time, inter-networking is ensured by the mobility management entity (MME), which communicates with both the home subscriber server (HSS) and the authentication, authorization, and accounting (AAA) unit. Further details on the handover procedure implementation between 3GPP and non-3GPP networks are given in [44].

## 3. Equipment and Scenario Description

This section describes the multi-RAT robotic platform and the scenario of interest for LHO evaluation.

### 3.1. Multi-RAT Robotic Platform

Here, the multi-RAT robotic platform used to evaluate our LHO scheme is presented. It can carry up to four kilograms of payload and reach 7 km/h speed. It is currently capable of performing remote operations in indoor environments for approximately two hours without recharging. The installed hardware allows us to detect obstacles located half-a-meter away from the vehicle, which in combination with the data obtained from the on-board camera makes it possible to implement capabilities for autonomous driving.

Our vehicular platform is equipped with cmWave (WiFi) and mmWave (WiGig) interfaces in order to provide multi-connectivity features. Wi-Fi interface is SL-1506 dongle working on RT5370N chip and implementing the standard 802.11n. The module uses closed-loop power control with an output power range between 2 and 18 dBm conveyed via a single 2 dBi omnidirectional antenna. As we used a Linux-based operating system, the communication rate adaptation was driven by the Minstrel algorithm, which is part of the mac80211 kernel subsystem [45]. In the case of mmWave connection, the vehicular platform is equipped with a Mikrotik wAP 60G client station based on the Qualcomm QCA6320 WiGig module with maximum Tx power of 21.67 dBm. The module is further provided with a 6×6 phased array antenna with a maximum gain of 13.5 dBi.

**Figure 2:**
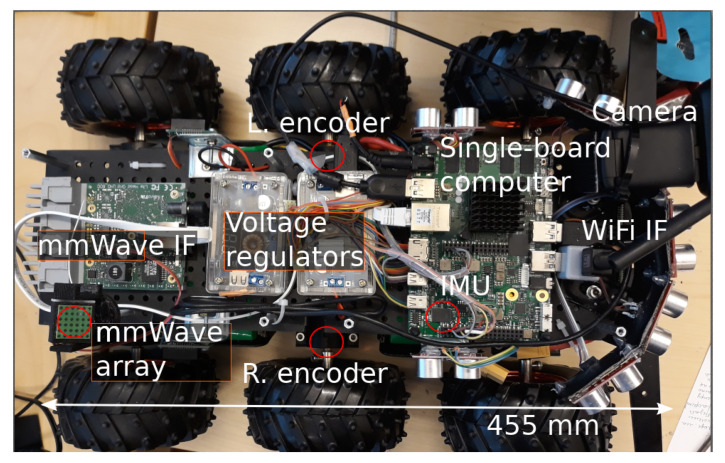
Prototype photo (disassembled) with notes.

At the core of the platform is an UDOO x86 single-board computer. In particular, the two motors of the vehicular platform are controlled via a RoboClaw motor controller connected to the UDOO board. Further, the RoboClaw has a power connection to external battery packs and a voltage regulator. A photo detailing the components of the platform is shown in Figure 2 and the technical details and features of the platform are summarized in Table 2. The platform can also operate in manual mode, where the vehicle is controlled by the operator remotely. Hence, the operator is monitoring the video stream provided by the camera installed on the front of the vehicle.

The platform allows us to collect various types of statistics. First, we monitor the communication performance by analyzing signal strength, end-to-end packet latency, and throughput data. For the installed WiGig transceiver, we also collect antenna array configuration statistics provided by the application programming interface (API) of the device. On top of that, UDOO board has an integrated six-axis sensor, which allows us to monitor the heading of the vehicle. Further, the motors of the driving wheels have magnetic encoders, which make it possible to estimate the location of the vehicle in relation to the starting position.

### 3.2. Scenario of Interest

**Figure 3:**
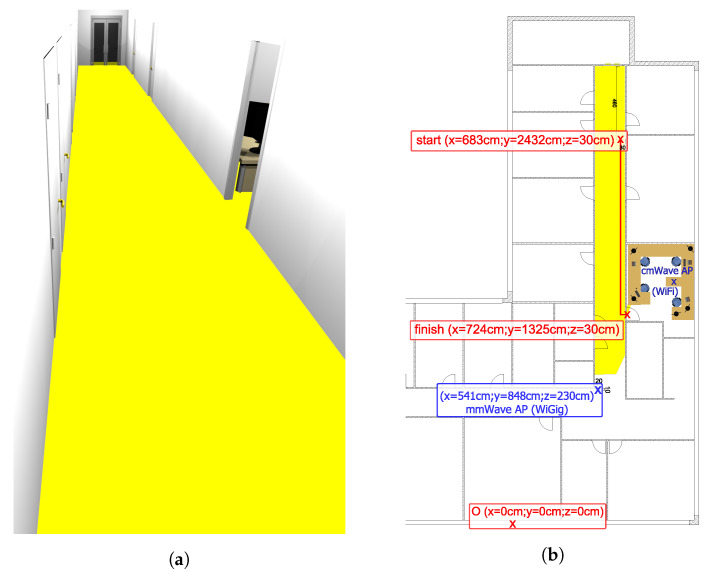
Test scenario 3D model and layout. (**a**) The 3D model of the corridor. (**b**) Test scenario layout combined with LoS map.

The scenario for evaluating the LHO scheme is presented in Figure 3 where a typical office setting is employed to mimic an industrial environment. The corridor, where the test was held is 3×2.5 m wide/high. The offices are separated from the corridor with 10 cm wide partition walls. Inside the office, the WiFi AP is installed on a 1.5 m height (see Figure 3). Along the office walls there are installed three wooden tables, four metal chairs and two cupboards, which do not obstruct the LoS condition between robot and WiFi nor robot trajectory during the test.

In terms of the robot trajectory, the starting location was 15 m away from the mmWave AP that is attached to the ceiling and tilted 15 degrees down, to provide stable coverage to the robot in the LoS conditions. The LoS coverage of the mmWave AP is modeled in Blender, as is discussed in Section 4.1 and shown in Figure 3a with the viewing angles corresponding to the location of the AP and its antenna array configuration span. The WiFi is located inside the office, in the NLoS state w.r.t. the initial location of the robot. It is noteworthy that there exists only one mmWave AP (WiGig) and one cmWave AP (WiFi) within the considered environment, and we tried to minimize the existing interference from other WiFi APs operating in the same area by choosing the least used frequency channel. The overall robot trajectory is as follows. After the first 10 s of initialization (when the vehicle remains stationary), the robot starts moving along the corridor with the constant velocity of approximately 0.5 m/s, until it reaches the entrance to the office, in which the WiFi is located, see Figure 3b. Further, it turns left and moves into the office (entering the mmWave NLoS area). In the end, the robot drives one meter inside the office and stops. All in all, the core objective of this work is to maximize the radio connection with the mmWave AP for an augmented throughput, while switching to the WiFi in the non-ideal condition for enhanced link robustness in the industrial environment.

Last but not least, we emphasize that the proposed handover scheme works the best in static environments, where the scenario layout either changes rarely, or changes are periodic and predictable. If there is a need to consider dynamic scenario reconfiguration, the LoS map of the environment should be modified accordingly, which certainly increases the overall complexity. An example of an environment with predictable geometry dynamics could be a fully automated factory or storage/production hall.

## 4. Enabling Multi-RAT Indoor Handover

In this section, the method of constructing the 3D indoor environment as well as the LoS map w.r.t. the mmWave AP is provided. Inheriting the ideas from [35,46], the developed GBP algorithm is then presented and formulated. Finally, the proposed handover scheme is described and discussed.

### 4.1. Determining mmWave LoS

Here, we describe the method [47] of building the LoS map of the mmWave AP within the considered environment. The main purpose of this method is to construct a 3D model of a particular environment in order to predict the LoS state w.r.t. the mmWave AP at any specific locations. In particular, the necessary steps are given as follows.

1. Building 3D model. The first step is to obtain a considered environment in the 3D modeling software that provides sufficient details to reflect all the objects that obstruct the LoS path between the objects of interest (e.g., the mmWave AP and the robot).
2. Identifying 2D plane. In the 3D model, we add a plane on the height corresponding to the device antenna. The surface of the plane in the 3D environment represents the set of all possible points where the device antenna can be situated. For further image processing simplification, the plane is uniformly colored, while the choice of its material results in the absence of shadows and reflections (illustrated in Figure 3a).
3. AP point of view (PoV) LoS map. A 2D render from the AP’s point of view is used to produce a “warped” map (shown as Figure 3a). The viewing angles are set to the antenna parameters of the AP.
4. Affine transform. The final step is to perform a geometric transformation to the projected PoV map in order to eliminate the z-coordinate as well as restore the proportions such that the orthogonal coordinate system (top view) of the LoS map of the area is obtained. In particular, the transform is carried out by applying three anchor points, which have been marked with the red cross shown in Figure 3b. It is important to note that the red line shown therein is employed as the ground truth of the robot trajectory in the simulation campaign (see Section 5.1), which is different from the ground truth of the robot trajectory in the experiments that is not measured and therefore remains unknown.

The red line plotted in Figure 3b is applied as the robot trajectory for simulation-based study on the RSSI, AoA and proposed positioning algorithm. The information provided by the LoS map (such as the LoS–NLoS border) is first integrated in the robot, and then utilized to decide whether the robot resides in the LoS region w.r.t. the mmWave AP for the experimental evaluation of the proposed handover scheme, which will be discussed in Section 4.3.

### 4.2. Proposed Positioning Algorithm

**Figure 4:**
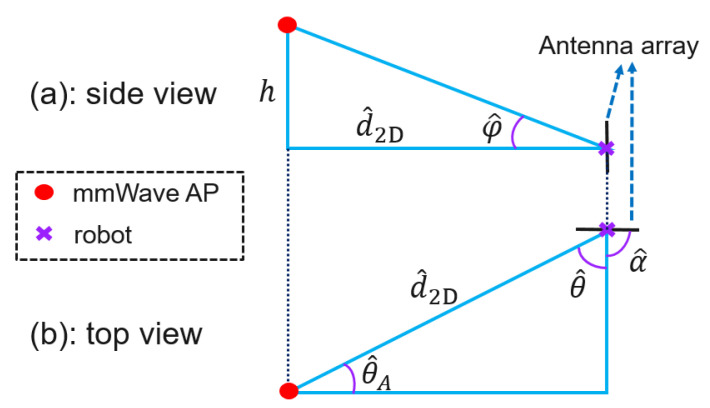
Illustration of geometric relationships between the robot and mmWave AP from two points of view.

Since the proposed positioning algorithm is geometry-based, we provide an illustration of the geometry relation between the mmWave AP and the robot in terms of the side view and top view in Figure 4, in which the GBP algorithm is constructed. Specifically, the mmWave AP is represented by the red dot, while the purple cross marker denotes the robot. Further, the antenna array deployed on the robot is highlighted by the black solid line on top of the purple markers. It is important to note that since the positioning is implemented at the robot side, the antenna array of the mmWave AP is therefore omitted. In addition, we denote the noiseless elevation and azimuth AoA at the robot side as φ and θ, and the true orientation of the antenna array (in the azimuth plane) on the robot after the transformation from the robot coordinates to the local 3D Cartesian coordinates [28] is α. In this work, only the array orientation in the azimuth plane (horizontal plane) has to be considered because the elevation orientation of the array remains vertical to the ground as the robot moves along the trajectory. Further, ^ denotes the noisy measurement of the corresponding noiseless quantity. By solving the geometrical relationship between the mmWave AP and the robot shown in Figure 4, the GBP algorithm is described in Algorithm 1, where the inputs contain the AoA measurements, $\hat{\varphi }$ and $\hat{\theta }$, and the orientation measurements $\hat{\alpha }$. Additionally, the GBP requires the location information of the mmWave AP to complete the radio-positioning for the robot. It is noteworthy that the time index is omitted in Algorithm 1 due to the independence between the location estimates of the adjacent time instants.

Today, there are various methods to estimate the robot location, e.g., the extended Kalman filter (EKF) [31,34,35,48] or the theoretical positioning error bound [27,49]. Here, we propose and apply the GBP for robot positioning due to three major reasons. The first one is the limited availability of LRMs. Given the considered scenario, there is only one mmWave AP available for acquiring the LRMs. For positioning with two unknowns (i.e., x−,y− components of a 3D coordinate), most of the existing algorithms require at least three APs. The second reason is the algorithm complexity. In order to reduce battery usage during positioning, the applied positioning algorithm needs to be less computationally complex. Comparing with the EKF, the GBP algorithm was shown to have much lower computational complexity while providing reasonable positioning accuracy [35]. Third, the main target of this work is not to compare different positioning algorithms but to acquire location awareness for better handover performance. Generally, a better positioning performance is directly related to a better LHO performance; however, as we discuss in Section 5.2, the LRMs obtained by the robot is too coarse to be used for accurate positioning by any positioning algorithms.
**Algorithm 1:** Geometry-based positioning (GBP) **Input**: $\hat{\varphi }$, $\hat{\theta }$, $\hat{\alpha }$, $x_{A}$, $y_{A}$
 **Output**:
$\hat{x}$, $\hat{y}$
1Compute the 2D distance between mmWave AP and the robot based on elevation AoA measurement $\hat{\varphi }$ and the known antenna height difference *h*
$${\hat{d}}_{2D}=h/\tan\left(\hat{\varphi }\right)$$2Convert the azimuth AoA measurement $\hat{\theta }$ at the robot to the angle of departure (AoD) ${\hat{\theta }}_{A}$ at the mmWave AP taking into account the array orientation measurement $\hat{\alpha }$ of the robot $${\hat{\theta }}_{A}=\pi -\left|\hat{\theta }-\hat{\alpha }\right|.$$3Calculate the robot location based on ${\hat{d}}_{2D}$ and ${\hat{\theta }}_{A}$
$$\left[\begin{array}{c} \hat{x}\\ \hat{y} \end{array}\right]=\left[\begin{array}{c} x_{A}+{\hat{d}}_{2D}\cos{\hat{\theta }}_{A}\\ y_{A}+{\hat{d}}_{2D}\sin{\hat{\theta }}_{A} \end{array}\right]$$

### 4.3. Proposed Handover Scheme

**Figure 5:**
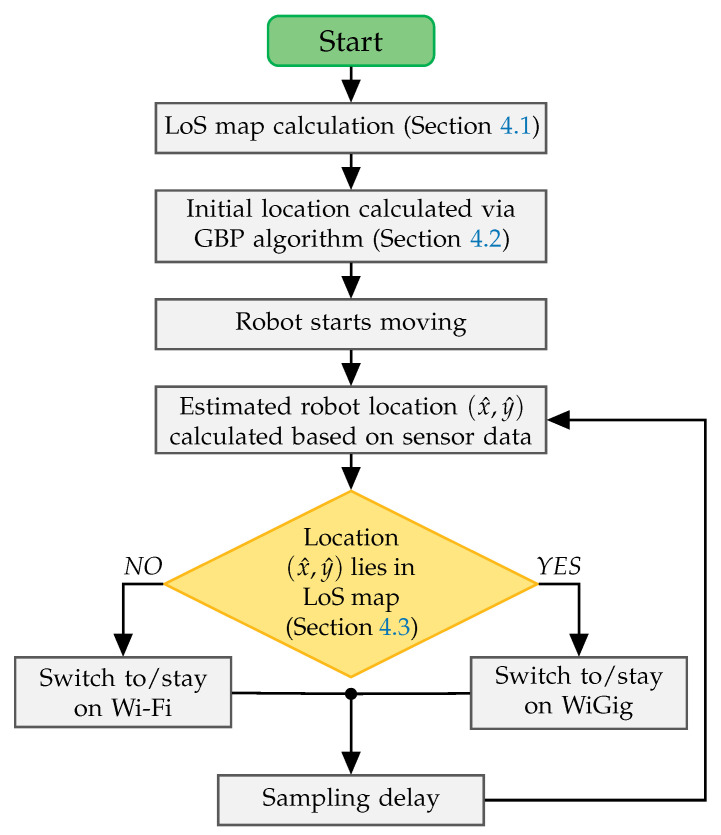
Procedures of the roposed LHO scheme.

The proposed handover scheme, LHO that is illustrated and described in Figure 5, is in general based on the knowledge of estimated robot locations and the potential LoS zone w.r.t. the mmWave AP location. In particular, the LHO is initiated whenever the robot enters/exits the mmWave NLoS area. To predict and execute the handover, the robot compares its current estimated location with the handover location on the LoS map of the environment that is shown in Figure 3. For instance, if the robot starts off in the LoS area, the LHO is triggered only if the estimated robot location no longer lies on the LoS map, in which case, a switch from mmWave RAT to WiFi shall occur. The performance of this scheme depends on multiple factors including the positioning accuracy, the presence of moving obstacles, and the robot speed. To improve the handover efficiency, one can take into account the robot trajectory, the channel statistics (measured signal level) and the sensor data (e.g., images/video, pseudo-range measurements).

In addition, traditional RSSI hysteresis and time triggers may be used to eliminate possible ping-pong effects [10]. However, in this paper we use a “proof of concept” scheme, which only takes into account the current estimated location of the robot, and triggers a handover when the robot crosses the LoS-NLoS border. Assuming that we know the map of the building and the LoS border, together with the current location of the robot, the task of predicting if the robot is in the LoS area becomes in checking if it is located inside the polygon of an arbitrary shape, i.e., points in polygon (PiP). Since the observed polygon can be non-convex, the most efficient way to solve the problem is via a ray intersection method with the complexity of O(N), where *N* represents the number of edges in the considered polygon [50]. In order to decrease the computing power requirements, the LoS area polygon should be calculated separately (using e.g., Blender) for each mmWave AP in the deployment scenario, while the checking algorithm should be applied only for the currently used AP.

The complexity and the feasibility of our scheme, therefore, depend on the complexity and the accuracy of the employed location estimation algorithm together with the complexity of the chosen PiP algorithm. As in the conventional schemes, the resultant computation intensity of the location-aware solution depends on the status (location) updates frequency. The implementation of the LHO scheme requires the storing of pre-calculated LoS maps and the realization of a mechanism to switch the packet flow from one RAT to another. In the paper, we compare the performance of the proposed handover scheme with a traditional RSSI-based approach. The RSSI-based handover is triggered when the WiFi RSSI is higher than the mmWave RSSI plus a 3 dB margin, for the duration of three consecutive measurements. Herein, the 3 dB hysteresis margin is set according to the recommendation made in [11]. It serves as the handover threshold throughout the overall handover process. Furthermore, the handover is implemented in a network-layer using “soft handover” approach—the data flow switching is enabled by changing the destination IP address, which triggers the routing table update and changes the physical interface (WiGig or WiFi TX) used for the transmission. In other words, the association (signaling) with appropriate AP (WiGig or WiFi) is not discontinued in order to record the appropriate statistics since it is connected to both APs at the same time. However, the implementation of the proposed algorithm could be realized using “hard handover” approach as well.

In terms of the fairness of the comparison between the two considered handover scheme, we provide the following elaborations: in general, both the RSSI-based handover and the LHO belong to the context-aware handover scheme. Therefore, the two handover schemes are different in terms of the utilized context for handover prediction and triggering. In [23], various context-aware handover schemes are discussed and compared. In particular, some works utilized the positioning system, such GNSS as the context parameter, whereas others measured the RSSI values and used them as the context parameter. In a way, the comparison between RSSI-based handover and LHO (in the same environment) is fair enough, since they merely utilized different context parameters for triggering the handover procedure. Nevertheless, LHO is in general more difficult to implement than the RSSI-based handover since it requires more information about the environment. Our contribution in this work is to demonstrate the potential performance gain when utilizing the estimated location information for the handover, hence the LHO, further enhancing the unification of positioning and communication technologies in the HetNets.

## 5. Obtained Results and Evaluation

In this section, the performance of both the simulation-based positioning algorithm and the experiment-based handover scheme is presented and discussed.

### 5.1. Simulation-Based Positioning Accuracy

**Figure 6:**
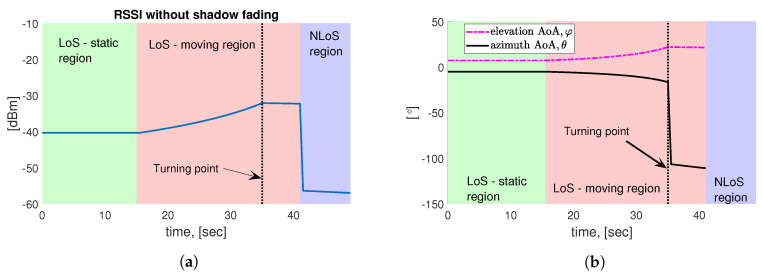
Simulation-based numerical characterization as a function of time along the simulated robot trajectory (see Figure 3b). (**a**) The received signal strength indicator (RSSI) applying InH office pathloss model ([52] Table 7.4.1-1); (**b**) Noiseless AoA with respect to mmWave AP.

First, noiseless elevation and azimuth AoA with respect to the mmWave AP together with the RSSI (at the robot) without shadow fading are plotted in Figure 6 for the comparison with the acquired experimental results, as will be shown in the next subsection. Specifically, the robot trajectory is divided into three segments, which are marked with different colors: first, the robot remains static in the light green region performing operational system initialization. Thereafter, it moves toward the office entrance (the light red region), where the robot makes a turn (black dashed line), and drives inside the office (toward the NLoS region w.r.t. the mmWave AP, i.e., the light blue region). On its way into the office, the robot passes the LoS-NLoS border, where the RSSI value significantly drops.

**Figure 7:**
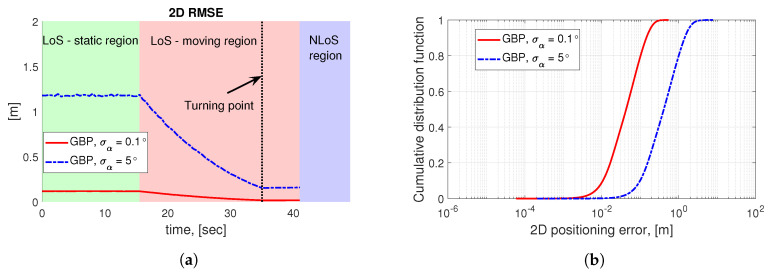
Positioning performance via GBP along the robot trajectory in Figure 3b over 2000 trials in the simulation campaign. It is noteworthy that the timeline of Figure 6a is slightly different than that of Figure 8 and Figure 9, which are obtained from the experiment. The reason lies in the fact that the ground truth of the robot trajectory is unknown in the experiment but is defined in the simulation campaign. (**a**) 2D RMSE as a function of time via simulation. (**b**) CDF of positioning error via simulation.

The positioning accuracy via a numerical simulation shown in Figure 7 is characterized by the 2D root mean square error (RMSE) and the corresponding cummulative distribution function (CDF) of the 2D positioning error along the considered robot track within the LoS region (see the red curve in Figure 3b). Further, the applied parameters of the simulation are summarized in Table 3, where the utilized pathloss model refers to the 3GPP indoor hotspot (InH)-office LoS scenario. In our simulation campaign, the Cramér-Rao lower bound (CRLB)-based LRMs ([33] Ch. 3), i.e., the elevation and azimuth AoA measurements based on the RSSI (see Figure 6a) are generated according to the methods in [35], which are then utilized as the input LRMs ($\hat{\varphi },\hat{\theta }$) of Algorithm 1 to obtain the corresponding theoretical accuracy in Figure 7. It is worth pointing out that, in Figure 7a, the positioning performance gradually becomes better in the light red region before the turning point, this is due to the fact that the robot is moving towards the mmWave AP, the RSSI level becomes higher (as shown in Figure 6a) which leads to more accurate AoA measurements that yields lower positioning errors.

The array orientation measurement $\hat{\alpha }$ is assumed to be corrupted by an un-biased Gaussian error with the standard deviation of $\sigma_{\alpha }$ that is set to two different values in the simulation campaign. Specifically, the curves in Figure 7 manifest the difference in terms of the obtained positioning accuracy at two different orientation error statistics ($\sigma_{\alpha }=0.1^{\circ }\mathrm{or}\;5^{\circ }$ [51]), which reflects the impact to the LRMs that stems from the stable or coarse orientation measurements obtained from a six-axis sensor on the robot. As it is observed in Figure 7, the positioning accuracy by GBP is generally higher for a smaller orientation error statistics $\sigma_{\alpha }$, as well as when the robot approaches the mmWave AP owing to the fact that for a given AoA measurement error, the robot suffers a smaller positioning error when it is closer to the mmWave AP. In other words, the AoA measurement error is translated into a larger/smaller positioning error at a larger/smaller UE-AP distance. It is also worth noting that the RMSE curve stops when the robot enters the NLoS region, thus indicating the fact that the communication with the mmWave AP is discontinued when the LoS is blocked.

### 5.2. Experiment-Based Handover Performance

In this experiment, we considered a user-centric handover procedure; hence, the serving entities (mmWave and cmWave APs) are used only to provide the necessary data for the robot to make a handover decision. While on a network-scale, the experiment may be envisioned as simply providing WiFi accessibility, handover targeting the preservation of appropriate data session continuity is carried out on the user side.

**Figure 8:**
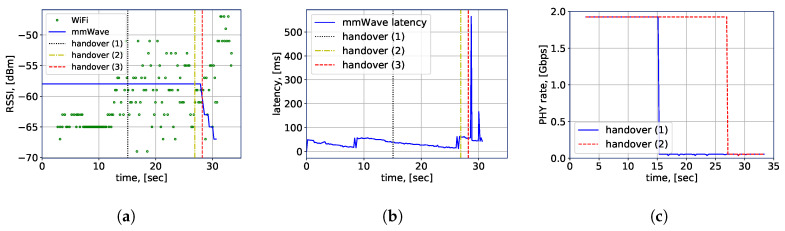
Measurements collected by the robot at the test scenario, (**a**) RSSI measurements; (**b**)WiGig latency measurements; (**c**) physical layer (PHY) rate.

The connection-related performance is displayed in Figure 8, where the horizontal axis represents the timeline of the handover experiment starting from 0 s. With the black, yellow and red vertical lines, the time of the RSSI-based (1), location-aware (2) and preferred (3) handover schemes are indicated, respectively. It is noteworthy that the handover (3) does not correspond to any practical handover schemes, it indicates the preferred handover location that maximizes the throughput while maintaining the link robustness throughout the whole considered robot trajectory in the experiment. Specifically, the RSSI measurements are shown in Figure 8a. It can be observed that the RSSI-based handover scheme switches to mmWave too early, while the location-based scheme is triggered just before the signal strength from the mmWave AP drops. Here, it should be mentioned that the RSSI-based handover performance is displayed for a particular measurement set and is limited by high variations of the WiFi RSSI in the NLoS environment. While adjustment of the hysteresis and time margins might improve the efficiency of the scheme in question, it is more complicated to achieve optimized performance due to the multi-RAT nature of the considered scenario. Similarly, in Figure 8b it is visible that the location-based scheme makes a switch just before the latency issues begin on the mmWave link; hence, it offers better performance than with the RSSI-based handover, which provides higher average throughput.

For a clear demonstration of the performance difference, we summarize the achieved physical layer (PHY) rate of the robot by both of the considered handover schemes in Figure 8c, in which, we refer to handover (1) as the RSSI-based scheme and handover (2) as the LHO scheme, respectively. It is evident that the maximum rate is achieved when the robot is connected to the mmWave AP, whereas the minimum case is acquired by the WiFi connection. More importantly, our experimental results indicate that over the considered time duration, the handover (2), i.e., the LHO, achieves nearly twice as high average rate (1.523 Gbits/s against 0.806 Gbits/s) than the handover (1) owing to location awareness that maximizes the connected time to the mmWave AP that provides a higher bandwidth, hence a higher throughput. Meanwhile, the link robustness is satisfactory as well since the vertical handover to WiFi was performed before the robot entered the NLoS region w.r.t. the mmWave AP.

Ideally, the robot should keep using the mmWave technology as long as possible, due to its higher throughput and lower latency. An early switch to WiFi affects not only the achieved throughput but also delay and jitter, which may become unsatisfactory for delay-sensitive applications, such as telemetry or high-resolution video streaming. Even though the performance of location-aware handover is better than a particular realization of the RSSI-based scheme, it is still not as efficient as the performance of handover scheme (3), the “preferred” option (illustrated as the red line in Figure 8a,b). However, delaying a handover may lead to high packet losses and potential disconnections due to small LoS–NLoS region on the border of the mmWave coverage areas. For example, it is observed in Figure 8a that after approximately 30 s the mmWave data are not collected due to a connection loss.

**Figure 9:**
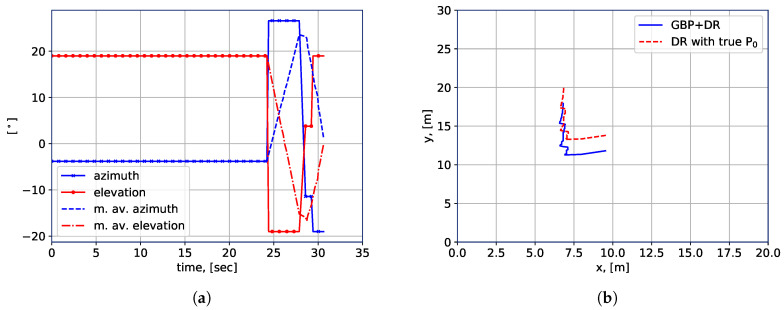
Practical implementation of location estimation algorithm, (**a**) WiGig beamforming variation; (**b**) Estimated and measured location.

Further, the performance of the LHO algorithm can be improved or degraded largely depending on the accuracy of the location awareness. In other words, when the positioning accuracy is high and reliable enough according to the environment and requirement, the LHO in general can achieve higher throughput than the RSSI-based handover. In the experiment, we apply the proposed GBP algorithm to compute only the initial location of the robot, thereafter, DR is applied for the location estimation of the rest of the trajectory. This is done because of the large errors of the measured AoA at the output of the antenna array, as shown in Figure 9a. By comparing Figure 9a,b, it is clear that the AoA measurements acquired by the robot do not reflect the changes of the AoA with a sufficient accuracy. Hence, standalone-GBP cannot provide adequate AoA accuracy for a reliable positioning accuracy and is therefore applied only to calculate the initial robot location. In terms of the reasons for the large errors of measured AoA, we provide the following elaborations. First, the employed mmWave equipment does not allow for continuous and “smooth” control over the array directivity. Instead, the used array has several fixed configurations, each corresponding to a particular vertical and horizontal direction of the main lobe. Each configuration covers approximately 10 degrees in the horizontal and vertical planes, which significantly decreases the accuracy of the LRMs, i.e., the AoAs. Second, we do not have access to the beam-searching algorithm, which makes it difficult to evaluate if the utilized configuration corresponds to a direct link or to a reflection from the wall or another object.

In Figure 9b, the estimated robot locations along the trajectory are plotted. For the DR-only case, we manually measure the true initial location of the robot, and the subsequent locations are measured by using robot IMU and wheel encoders data, i.e., the DR. In the “GBP+DR" case, the GBP (see Algorithm 1) is utilized to calculate the initial location, while the rest of the algorithm operates in the same way as in the DR-only case. The array configuration data received from the mmWave AP allows us to estimate the initial location of the robot with approximately one meter precision, which is sufficient to make adequate handover decisions in our case. However, we did not use the standalone-GBP method to estimate the location due to the precision-related issues as discussed above. It should also be noted that the precision requirements depend on the building layout and the robot dimensions.

In summary, our experiments and the corresponding results validate the feasibility of the LHO for mobile communications in a multi-radio environment, the observed performance gap of the considered handover schemes can be exploited for a more efficient handover scheme design enabling a self-organized communication system with autonomous vehicles in mmWave-ready HetNets, where both the link robustness and data throughput can be reliably guaranteed and sufficiently provided.

## 6. Conclusions

In this paper, we presented the enabling methods and the experimentally-oriented feasibility study on the LHO utilizing a multi-RAT robotic platform in an indoor HetNets environment with the target to achieve enhanced link robustness as well as augmented throughput, thus enabling a self-organized communication system with autonomous vehicles. In terms of location awareness, we developed and presented the GBP algorithm for positioning the constructed robot. The corresponding positioning accuracy has been tested via simulations by utilizing the CRLB-level AoA measurements with the array orientation uncertainty taken into account.

In addition to the numerical analysis, an experimental measurement campaign was conducted with the multi-RAT robotic platform in order to justify the feasibility of the proposed positioning algorithm as well as the LHO algorithm. Despite the coarse AoA measurements, our experimental results confirmed that the “GBP+DR” method is capable of providing sufficient positioning accuracy, which is essential to perform the LHO. With the obtained location awareness, the handover was carried out more precisely before the robot entered the NLoS region. Owing to a longer LoS connection with the mmWave AP, the LHO can achieve twice higher throughput than the conventional RSSI-based handover scheme. During the experiments, two independent data streams were maintained: one to control the telemetry connection and another one to provide video feedback between the robot and its remote operator. In both cases, session continuity targeted by the handover procedure was preserved, although a noticeable lag was observed in the video stream.

Even though the performance of the LHO scheme is bounded by the practical limitations of the employed equipment, it was shown via simulations that the applied positioning approach is capable of offering accurate enough location estimates in realistic indoor environments, thus further improving the performance of the discussed LHO in a multi-radio environment. Four directions of future work are currently envisioned: (i) development of advanced algorithms for more accurate AoA estimation based on the received signals at the mmWave array on the robot; (ii) assessment of practical performance levels in different types of environments and under varying densities of multi-radio network deployments considering potential interference; (iii) study and investigation of a hybrid handover scheme integrating various metrics, such as RSSI and location awareness under different environments and scenarios; (iv) together with positioning, the sensing techniques will also be applied to produce a simulated environment for multi-RAT handover with efficient beamforming strategy, expanding the proposed algorithm well beyond the considered scenario.

## Figures and Tables

**Table 1:** Advantage and disadvantage of different RATs.

RAT	Advantages	Disadvantages
cmWave (WiFi)	High robustness to blockage; low diffraction loss	Low throughput due to limited bandwidth
mmWave (WiGig)	High throughput at LoS owing to large bandwidth	Low robustness to blockage; high diffraction loss

WiFi standard: IEEE 802.11n. WiGig standard: IEEE 802.11ad.

**Table 2:** Technical specifications and features of multi-RAT robotic platform.

Framework	Dagu Wild Thumper Chassis with 6 Wheels and 2 Motors
Computing unit	UDOO ×86
Operating system	Debian Jessie
Radio access technologies	MikroTik wAP 60G (mmWave) and 802.11n Wi-Fi transceiver
Battery	2×8000 mAh LiPo
Camera	Logitech C270 HD
Sensors	SEN-13959 distance meters and 2× quadrature wheel encoders and BMI160 six-axis sensor

**Table 3:** Utilized simulation parameters.

Parameter	Value
Carrier frequency	60.5 GHz
Signal bandwidth	2.16 GHz
Transmit power @ AP *	21.64 dBm
Max. array gain @ AP *	13.48 dBi
Max. array gain @ robot	13.48 dBi
Robot update interval	0.5 s
Pathloss model	InH-office [52]
Fast fading model	Rician distribution

* The “AP”s mentioned in the table refer to the mmWave AP rather than WiFi since the simulation is carried out to model the communication between the mmWave AP and the robot.
